# Learning Attentional and Gated Communication via Curiosity

**DOI:** 10.1155/2022/2951193

**Published:** 2022-04-26

**Authors:** Chuxiong Sun, Kaijie Zhou, Cong Cong, Kai Li, Rui Wang, Xiaohui Hu

**Affiliations:** ^1^University of Chinese Academy of Sciences, Huairou, Beijing 101400, China; ^2^Institute of Software, Chinese Academy of Sciences, Haidian, Beijing 100190, China

## Abstract

Due to the partial observability in decentralized multi-agent systems, communication is critical for cooperation. Furthermore, the ability to decide when and whom to communicate is important to achieve efficient communication. However, the existing methods are typically driven by extrinsic rewards. Hence, when the reward from environment is sparse, delayed, or noisy, the communication performance of these methods would be restricted. Furthermore, it would introduce additional difficulty named credit assignment when using extrinsic reward to train communication and sample policies together. To tackle these difficulties, we introduce the mechanism of intrinsic motivation from psychology to multi-agent communication. We hold the view that the observations with more uncertainty and curiosity are more valuable for communication. It can help agent find useful information from observations. It is a good complement to existing extrinsic driven methods. Concretely, at sending end, we learn a curiosity from local observations to model the communication importance. Then, we design a heuristic mechanism to prune unnecessary messages. It can solve the problem of when to communicate. Then, the ability to gate unnecessary message can reduce the cost and improve the efficiency of communication, which is important to apply to real-world scenarios. Furthermore, at receiving end, we utilize the intrinsic importance to differentiate information, which can be helpful for local decisions. It could solve the problem of whom to communicate. The ability to pay attention to useful information can efficiently improve the performance of communication behaviors. At last, we evaluate our method on a variety of multi-agent scenarios. The experiments of full communication demonstrate that the curiosity is capable to model the communication importance, and the results of gated communication further prove the conclusion.

## 1. Introduction

For human beings, communication is a crucial tool to promote the progress of civilization. Similarly, the ability to drive efficient communication has recently received growing interests in the literature of multi-agent reinforcement learning (MARL). Particularly in some real-world tasks and applications where agents are required to make decentralized decisions merely based on local observations, the ability to communicate would make further sense. Concretely, the ability to decide what to communicate is an important challenging step to drive multi-agent communication [1]. However, the full communication among all agents at every moment would introduce the large computation and communication cost, but the exchanged messages may not always aid in decisions. Hence, the ability to decide when to communicate based on observed information is another challenging step to achieve effective communication, especially in some scenarios where the communication resources (e.g., bandwidth) are restricted [2–7]. Furthermore, how to differentiate incoming messages and distinguish promising information from them at receiving end is another challenge to achieve efficient communication [2, 8, 9].

In this work, we learn a communication value from agents' local observations and propose a novel value-based multi-agent communication architecture. It is an expanded version of [10]. Concretely, the message in the proposed protocol encompasses the content of information, as well as the importance of the shared information. Agents would communicate to their teammates only when the information they observed is meaningful and promising; hence, the resources could be allocated to valuable information and unnecessary communication would be avoided. Furthermore, after receiving messages, agents would also differentiate the incoming information and pay attention to important messages, which can aid in cooperative behaviors.

The key challenge in our framework is how to measure the communicated values of local observations in a promising way. Although the literature, which directly learns a value to formulate the communicated importance of observed information, is largely absent, the existing communication protocols are hundred percentage driven by the environmental reward. Hence, it can be concluded as “*Communicate what rewards you*.” The mechanism is widely used and makes significant progress in multi-agent communication. However, it may not work when the extrinsic rewards are sparse or nondecomposable. To this end, we propose a mechanism named intrinsic motivated multi-agent communication in this work. We hold the view that the curious and uncertain observations are important for communication since it can help agents get more unknown information. The motivation of IMMAC can be presented as “*Communicate what surprises you*.” Furthermore, we notice that the proposed IMMAC enjoys good scalability. It could be a good complement to the existing methods driven by extrinsic rewards.

In order to verify whether intrinsic motivated communication can aid in cooperation, we evaluate our algorithm on various environments and compare it with various baselines and ablations. At first, we consider full communication and evaluate the performance of intrinsic value-based attention mechanism. The results show that intrinsic values are sufficient to motivate efficient communication behaviors. Then, we combine the intrinsic and extrinsic attention mechanism. The results indicate that the combination of them can enjoy the benefits of both worlds. It demonstrates that the intrinsic motivated communication could be a good complement to extrinsic motivated communication. Finally, we consider gated communication and apply an intrinsic value-based gating mechanism to prune useless messages. The results show that it can generate comparable results to full communication under limited communication, which once more proves that intrinsic value is an effective way to measure the importance of observed information.

Overall, our contributions can be concluded as following:

- A novel value-based multi-agent communication framework is proposed. The framework enables agents to encode local observations and decide when to communicate and how to distinguish promising information.
- A novel communication mechanism motivated by intrinsic values is presented. To the best of our knowledge, we are the first to introduce the mechanism of intrinsic motivation to multi-agent communication. Moreover, the curiosity of local observations is modeled through the paradigm presented in [11]. It can scale to decentralized execution since the prediction problem is randomly generated and the curiosity can be obtained when agents only have access to local observations during execution.
- An extensive evaluation on our method and a comprehensive comparison of state-of-the-art methods are implemented, and we demonstrate that intrinsic motivated communication is a feasible idea.

The rest organization of this work is described as follows. In Section 2, we firstly introduce the related works about decentralized multi-agent reinforcement learning. Then, we describe the concept of intrinsic motivation and introduce the popular methods of intrinsic rewards in reinforcement learning. In Section 3, we formally present the problem considering in this work. Concretely, we provide the formulation and notation of Dec-POMDP, centralized training with decentralized execution, and value function factorization along with intrinsic motivation. In Section 4, we introduce the details of the proposed method. Firstly, we describe the architecture of our intrinsic motivated multi-agent communication agent and propose how to model intrinsic importance in decentralized scenarios. Then, we show how to use the importance of observed information to drive efficient communication. At last, we provide the training details and the way to combine our methods and existing works. In Section 5, we demonstrate the effectiveness of our method. We introduce a variety of environments, scenarios, and baselines used in this work. Then, we report the results of attentional and gated mechanism, which prove that our method can drive efficient communication. In Section 6, we provide a conclusion of this work. Concretely, we conclude the idea, method, and experiment of our method. Then, we provide a concise comparison to existing methods.

## 2. Related Works

### 2.1. Multi-Agent Reinforcement Learning

Recently, deep reinforcement learning (DRL) has enjoyed great success in many scenarios such as Go, Atari, and Robotics. However, the most success has been restricted to single-agent environments and tasks. And the real-world scenarios often require DRL algorithms to control multiple agents. In order to apply the DRL algorithms to multi-agent paradigm, the following difficulties are required to overcome.

- *Scalability.* One way to scale DRL algorithms to multi-agent settings is to learn a centralized policy or value function to control multiple agents [12]. These methods simplify multi-agent reinforcement learning to a single-agent problem on joint observation and action space. However, these methods are hard to scale since the joint observation and action space would grow exponentially in the number of agents. In addition, centralized methods would introduce the problem named “lazy agent” [13] in which agents would be sacred and lazy to explore when teammates find efficient policies. On the other hand, learning independent policies is another way [14, 15]. The independent policies are easier to learn since it only considers local observations. However, the fully decentralized learning problem would be instable since each independent agent regards other agents' influence as a part of environment. Hence, the environment would be nonstationary from the perspective of individual agent since it is hard to distinguish the stochasticity of environment and the changing policies of teammates [16]. In order to solve this problem, the hybrid paradigm named centralized training with decentralized execution (CTDE) is proposed [17]. The hybrid framework can enjoy the benefits of both fully centralized and decentralized methods and recently becomes a standard and popular paradigm for multi-agent reinforcement learning. For example, MADDPG [18] follows the CTDE paradigm and proposes a novel training framework, where actors are required to make decentralized decisions and critics are augmented with additional information (e.g., the information of teammates and global states) to ease training.
- *Credit Assignment.* The MARL problems often require multiple agents to optimize a single team reward based on joint actions. Hence, agents may observe deceptive reward signals that are originated from teammates [13]. Due to the inability to distinguish agents' contribution to team reward, credit assignment becomes an important open problem for MARL literature. Learning value factorization networks is an efficient way to solve this problem. Concretely, VDN [13] assumes that the global value function can be factorized to the summation of local values and proposes a linear value decomposition architecture. QMIX [19] replaces the additive assumption with a monotonic constraint and proposes a nonlinear value decomposition network. However, VDN and QMIX can only address tasks that can be factorized and meet the assumptions. By transforming the value function to an easily factorized one, QTRAN [20] extends it to a boarder setting without considering such assumptions. Furthermore, the value factorized methods may lead to suboptimal policies since the constraints on value functions would limit the farsighted exploration [21]. In order to improve exploratory ability, MAVEN [21] introduces diverse value approximations by injecting a shared and parameterized latent variable to mixing network. QDPP [22] finds natural value decomposition by utilizing determinantal point process [23] to provide diverse models during training. On the other hand, a multi-agent actor-critic framework named COMA [24] introduces a difference reward to solve the problem of credit assignment. Concretely, COMA replaces one agent's action with a default one and computes the difference of received reward, then uses the difference as a counterfactual baseline to model the individual agent's influence to team reward. Furthermore, DOP [25] applies the idea of value decomposition to the actor-critic framework and proposes a scalable policy gradient method.
- *Communication.* The CTDE paradigm and credit assignment methods can introduce implicit cooperation by introducing information to the learning algorithms. A more direct way to aid in cooperation in multi-agent literature is to share observed information among agents. Typically, the symbols for communication are predefined and fixed during training [26–28]. However, the predefined architecture may restrict communication [2]. To this end, [1] propose an architecture named CommNet, which can learn continuous messages from local observations. By learning the interaction with environment, the continuous messages can accommodate to the dynamic environment and finally benefit decision-making. Indeed, the difficulty of extracting information and make decisions based on incoming messages has been largely resolved. Furthermore, in order to decide whether to communicate based on observed information; [2, 3] design a gating network to output a binary communicated action; [4] propose a heuristic mechanism, where the communication only happens when agents cannot make confident decisions; and [5–7] adopt a weight-based scheduler to control communication, it would only allocate communication resource to agents with important observations. However, the majority of mentioned works straightforwardly integrate incoming messages with equal weights. It means that agents treat each received message equally. The naive mechanism is nonsophisticated and intelligent agents should be able to identify important information from sundry messages. In order to distinguish incoming messages, [2] utilizes a bi-directional LSTM unit, which can ignore useless information to integrate incoming messages, and [8] uses a soft attention mechanism jointly generated by senders and receivers to compute the importance weights for each message. Furthermore, [9] achieves target communication by introducing two information-theoretic regularizers. [29] utilizes the influence of one agent to others to model the necessity of communication and applies a request-reply paradigm to decide whether to communicate to another agent. Among the mentioned communication protocols, our work mostly relates to [5–8] since they also leverage an observation-dependent weight to control communication. From the perspective of framework, [5–7] is designed to decide when to communicate, [8] is proposed to decide whom to communicate, but our work can be regarded as a combination of them that can enjoy the benefits of both worlds. Furthermore, our work radically differs from them by the method to represent the importance of observed information. We consider both intrinsic and extrinsic values to evaluate local observations. The communication in our work is motivated by extrinsic rewards as well as intrinsic curiosity and uncertainty.

### 2.2. Intrinsic Motivation

Our work is also relevant to the works of intrinsic motivation. The concept is originated from psychology. In contrast to extrinsic motivation arising from outside, intrinsic motivation refers to behaviors, which are driven by internal return [30]. In recent years, intrinsic reward was introduced to the domain of reinforcement learning to explore novel behaviors. A variety of state-dependent reward bonuses are proposed to measure intrinsic values [31–35]. At first, the most straightforward way is to use visited counts to reward novel states [36, 37]. However, the naive idea cannot scale to large-scale problems since it uses a table to record visited counts. [31] uses a density model over state space to estimate the pseudocount, and [35] proposes a hash count, which can simplify high-dimensional state space.

In this work, we introduce the mechanism of intrinsic motivation to the literature of multi-agent communication since how to measure the importance of local observations is also promising to achieve efficient communication and the intrinsic value is a good complement to the existing solutions. However, most existing intrinsic values cannot scale to the decentralized multi-agent tasks since agents only have restricted access during execution. We conduct a careful filtration and finally adopt the prediction error based on random network distillation (RND) [11] to measure the intrinsic values of local observations. The detailed methods to model intrinsic values and use intrinsic values to drive communicated behaviors are presented in the following sections.

## 3. Background

During execution, agents only have access to local observations but can communicate observed information to others. Furthermore, at each time step, each agent is required to make decentralized decision *a*_*i*_^*t*^=*π*(*o*_*i*_^*t*^, *c*_*i*_^*t*^; *θ*_*i*_). Then, the environment would transit to a new state *s*_*t*+1_ based on the joint actions (*a*_1_^*t*^,…, *a*_*n*_^*t*^) and each agent would receive a new local observations *o*_*i*_^*t*+1^. Furthermore, a team reward *r*_*t*_=*r*(*s*_*t*_, *a*_1_^*t*^,…, *a*_*n*_^*t*^) is given to all agents. During training, additional information such as global states of environment, behaviors, and trajectories of other agents is provided and the decentralized polices are centrally trained to achieve a common goal, maximizing discounted team reward ∑_*t*=0_^*h*^*γ*^*t*^*r*_*t*_. It conforms to the paradigm of centralized training and decentralized execution [13, 18, 19, 24].

### 3.1. Value Function Factorization

In order to solve the difficulty of credit assignment in Dec-POMDPs, recent works [13, 19, 20, 22] focus on value function decomposition, which leverages a mixing mechanism to learn the joint Q-values of each agents. Concretely, VDN [13] directly uses the sum of local value functions to represent the joint value function.

(1) $$\begin{array}{c} Q_{tot}\left(s_{t},a_{t}\right)=\sum_{i=1}^{n}Q_{i}\left(o_{i}^{t},a_{i}^{t};\theta_{i}\right), \end{array}$$

where *Q*_*tot*_ refers to the global value function and *Q*_*i*_ denotes the local value function of agent_*i*_.

QMIX [19] replaces the summation by a nonlinear combination, whereas the following constraints are required:

(2) $$\begin{array}{c} \frac{\partial Q_{tot}}{\partial Q_{i}}\geq 0,\forall a\in A, \end{array}$$

where *A* refers to the joint action space that consists of a set of available joint actions.

### 3.2. Intrinsic Motivation in Reinforcement Learning

There is a diversity of state-dependent intrinsic rewards designed to represent the novelty, curiosity, and uncertainty of state space. In practice, most intrinsic values can be divided into two classes: count-based methods [31, 35–39] and prediction-error methods [33, 34, 40–42]. The count-based methods straightforwardly use the visited counts to model the novelty of states.

(3) $$\begin{array}{c} r_{i}^{t}=n{\left(s_{t},a_{t}\right)}^{\left(-1/2\right)}, \end{array}$$

where *r*_*i*_^*t*^ refers to the intrinsic reward, and *n*(*s*_*t*_, *a*_*t*_) is the visited count of *s*_*t*_ and *a*_*t*_.

The key insight of count-based methods can be concluded as lower frequency means higher novelty. Furthermore, the novel states typically encompass important information, which are uncertain to agents.

On the other hand, the prediction-error methods would formulate a state-dependent prediction problem, such as predicting next state given current state and action.

(4) $$\begin{array}{c} s_{t+1}^{\prime }=f^{\prime }\left(s_{t},a_{t}\right), \end{array}$$

where *s*_*t*+1_′ refers to predicted next state. Then, the prediction errors ‖*s*_*t*+1_ − *s*_*t*+1_′‖^2^ are used to represent the uncertainty through state space.

The modeled intrinsic values are promising to drive exploratory behaviors in the literature of single-agent reinforcement learning when extrinsic rewards are sparse or deceptive. In this work, we introduce the intrinsic motivation to encourage communicated behaviors for MARL. We hold the view that it would be a good complement to the existing communication works, which only consider extrinsic rewards to evaluate the importance of observed information.

## 4. Method

### 4.1. Architecture

**Figure 1:**
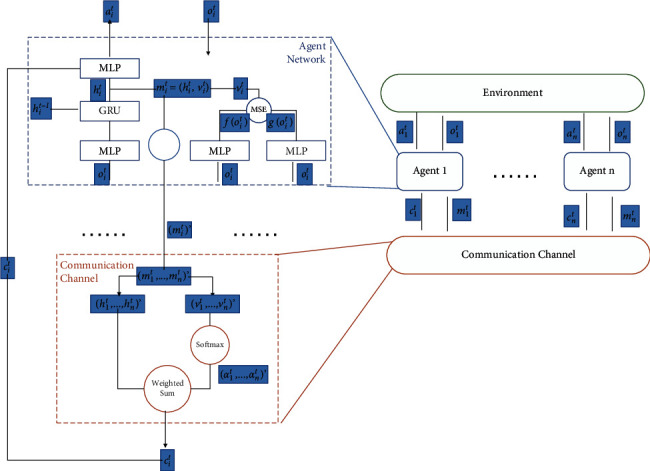
The detailed architecture of IMMAC. At time step *t*, agent *i* gets local observation *o*_*i*_^*t*^ and shares observed information *m*_*i*_^*t*^ to other agents, then receives the integrated messages *c*_*i*_^*t*^ from communication channel and produces action *a*_*i*_^*t*^ for interacting with environment. More particular, the policy network takes the local observation *o*_*i*_^*t*^ and aggregated message *c*_*i*_^*t*^ as input and outputs the action values for available actions. Intrinsic value network takes *o*_*i*_^*t*^ as input and outputs an observation-dependent value *v*_*i*_^*t*^, which is used to distinguish important local observations.

As illustrated in Figure 1, our framework encompasses a partially observed environment, an attentional communication channel, and *n* independently controlled agents. At time step *t*, each agent *i* would receive a local observation *o*_*i*_^*t*^ from environment and an integrated message *c*_*i*_^*t*^ from communication channel. Since agents have no access to the global state *s*_*t*_ during execution, the information sharing would be helpful for cooperation. Concretely, each agent consists of a policy network along with an intrinsic value network and a gating mechanism. The policy network encompasses an observation encoder and an action generator. The observation encoder is implemented as a 1-layer multilayer perception (MLP) and a 1-layer gated recurrent unit (GRU) [43]. It takes local observations as input and is responsible for encoding the local observation histories. Then, the embeddings *h*_*i*_^*t*^ are concatenated with the incoming messages *c*_*i*_^*t*^ and serve as an input to action generator.

Furthermore, the intrinsic value network, which maps observations to communication values, is responsible for measuring the intrinsic importance of observed information. In order to model importance of the shared information, we randomly formulate a prediction problem related to agent's local observations. The prediction problem is defined by a target network *f* : *O*⟶*R*^*k*^, which consists of a 2-layer MLP. The target network is randomly initialized and fixed during training so that the prediction problem does not have dynamics. The predict network *g* : *O*⟶*R*^*k*^ implemented as a 3-layer MLP is designed to answer the prediction problem. It is trained on experience collected by all agents and learned by minimizing the following MSE ‖*f*(*x*) − *g*(*x*)‖^2^. We aim to leverage agent's ability to make predictions from local observations (i.e., the ability to understand *o*_*i*_^*t*^) to model the novelty and uncertainty of observed information. In other words, agents typically cannot make precise predictions on the observations with novel and uncertain information. Such prediction errors tend to be large when the observations are novel, and the errors would decrease as the agent collect more experience similar to *o*_*i*_^*t*^. Moreover, the novel observations typically encompass important information, which can help agents understand environment. Therefore, it is promising to share novel and uncertain observations to teammates. Similar to communicate rewarding observations, sharing novel observations is also an effective direction for communication. It enables agents to distinguish whether the observed information is important.

The parameters of policy network and intrinsic value network respectively refer to *θ*_*p*_=(*θ*_*e*_, *θ*_*a*_), *θ*_*v*_=(*θ*_*f*_, *θ*_*g*_), and all parameters are shared among agents. Furthermore, our architecture also encompasses a gating mechanism, which is responsible for pruning useless messages, and an attentional mechanism, which is designed to integrate incoming messages. The communicated details are provided in next subsection.

### 4.2. Decentralized Communication and Execution

The shared information *m*_*i*_^*t*^ in this work consists of two parts:

(5) $$\begin{array}{c} m_{i}^{t}=\left[\overset{\text{information}}{\overset{︷}{h_{i}^{t}}},\underset{\text{importance}}{\underset{︸}{v_{i}^{t}}}\right], \end{array}$$

where *h*_*i*_^*t*^ is extracted from observed information and *v*_*i*_^*t*^ describes how important is the information. The observed information and importance are, respectively, generated from the policy network and intrinsic value network.

(6) $$\begin{array}{c} h_{i}^{t}=\pi_{{\text{layer}}_{2}}\left(o_{i}^{t}\right), \end{array}$$

where *π*_layer_2__ denotes the output of the second layer of policy network.

(7) $$\begin{array}{c} v_{i}^{t}={\left\|f\left(o_{i}^{t}\right)-g\left(o_{i}^{t}\right)\right\|}^{2}, \end{array}$$

where *f* and *g*, respectively, denote the predict network and target network mentioned in subsection 4.1.

At first, each agent would generate the information and intrinsic importance for communication. Further, each agent would pass the information to a gating model. The gating model can avoid unnecessary information sharing. Concretely, it equip agents the ability to decide whether to communicate or not. In this work, the gating model is implemented as a heuristic mechanism based on intrinsic importance.

(8) $$\begin{array}{c} g_{i}^{t}=\left\{\begin{array}{cc} 1, & v_{i}^{t}\geq \beta ,\\ 0, & v_{i}^{t}<\beta , \end{array}\right. \end{array}$$

where *g*_*i*_^*t*^ is a binary information that is used to decide whether to communicate. Concretely, when the importance of observed information is larger than a threshold *β*, the agent_*i*_ would share the information among agents. Otherwise, agent_*i*_ would not communicate with other agents. Hence, the gated information can be generated by the following equation:

(9) $$\begin{array}{c} h_{i_{\text{gated}}}^{t}=h_{i}^{t}\cdot g_{i}^{t}, \end{array}$$

where *h*_*i*_ted__^*t*^ denotes the gated information. The ability to decide when to communicate is important to communication efficiency since in many real-world scenarios, the resources for communication are limited, so it is promising to differentiate observed information and share the important of them.

Then, the gated information from agents would be sent to a shared channel. The channel is designed to integrate incoming information. We implement an attentional mechanism in this work. Concretely, we directly use the intrinsic importance to represent the attention weights so that the information with large intrinsic importance would be payed more attention.

(10) $$\begin{array}{c} \left(\alpha_{1}^{t},\ldots ,\alpha_{n}^{t}\right)=\text{softmax}\left(v_{1}^{t},\ldots ,v_{n}^{t}\right), \end{array}$$

where *α*_1_^*t*^,…, *α*_*n*_^*t*^ denote the attention weights.

(11) $$\begin{array}{c} c_{i}^{t}=\sum_{i=1}^{k}\alpha_{i}^{t}h_{i_{\text{gated}}}^{t}, \end{array}$$

where *c*_*i*_^*t*^ is the information aggregated from all agents.

We notice that the introduced attentional mechanism can help agent differentiate important information when integrating incoming messages. It can help agent get more useful information. Finally, the integrated information would broadcast to each agent and fed into the policy network to help make better decisions.

(12) $$\begin{array}{c} a_{i}^{t}=\pi_{i}\left(o_{i}^{t},c_{i}^{t}\right), \end{array}$$

where *a*_*i*_^*t*^ denotes the local decision and *π*_*i*_ refer to the policy of agent_*i*_.

### 4.3. Centralized Training

During training, additional information such as global states is available. However, the training of intrinsic value network is completely intrinsic. It does not rely on any extrinsic and task-specific reward signals. Concretely, the parameters of intrinsic value network are updated using the following MSE:

(13) $$\begin{array}{c} L_{\theta_{v}}={\sum_{t}\sum_{i}\left[f\left(o_{i}^{t};\theta_{f}\right)-g\left(o_{i}^{t};\theta_{g}\right)\right]}^{2}, \end{array}$$

where *θ*_*f*_ is used to formulate prediction problem and fixed during training, and *θ*_*g*_ refers to the parameters of predict network.

In addition, the policy network is trained by reinforce loss and extrinsic rewards.

(14) $$\begin{array}{c} L_{\theta_{p}}={\sum_{t}\left[y_{\text{tot}}^{t}-Q_{\text{tot}}\left(s_{t},a_{t};\theta_{p}\right)\right]}^{2}, \end{array}$$

where the optimal target values are computed using Bellman equation, *y*_*tot*_^*t*^=*r*_*t*_+*γ*max_*a*′_*Q*_*tot*_(*s*_*t*+1_, *a*′; *θ*_*t*_), and the joint value functions are combined using the paradigm presented in [19], *Q*_tot_=*F*(*Q*_1_(*o*_1_^*t*^, *a*_1_^*t*^; *θ*_1_),…, *Q*_*i*_(*o*_*i*_^*t*^, *a*_*i*_^*t*^; *θ*_*i*_), *s*_*t*_; *θ*_mix_).

### 4.4. Combination of Intrinsic and Extrinsic Motivated Communication

We introduce the mechanism of intrinsic motivation to encourage multi-agent communication. However, we do not regard the IMMAC as an alternative to the existing extrinsic motivated communication. We further hold the view that intrinsic motivated communication can be a good complement to existing extrinsic motivated communication, especially in the scenarios where extrinsic motivated communication does not work. The extrinsic and intrinsic motivation can be regarded as two senses separately sensing the environment and jointly aid in decision making. The combination of extrinsic motivation and intrinsic motivation may enjoy the benefits of both worlds.

In this work, we directly model an intrinsic value from local observations and propose a value-based framework to control communication. Obviously, the proposed framework is straightforward to combine with existing extrinsic weight-based communication strategies such as [4, 5, 7, 44]. Concretely, we denote the extrinsic communicated significance as *v*_*e*_^*t*^ and directly combine it with the proposed intrinsic importance.

(15) $$\begin{array}{c} v^{t}=\beta_{e}v_{e}^{t}+\beta_{i}v_{i}^{t}, \end{array}$$

where *β*_*e*_ and *β*_*i*_ are hyperparameters to balance the intrinsic and extrinsic values.

## 5. Experiment

In this section, the experiments are designed to investigate the following problems:

- Whether intrinsic values can be served as an effective motivation for communicated behaviors
- Whether the proposed intrinsic value-based framework can improve cooperative performance and efficiency
- Whether the straightforward combination of intrinsic and extrinsic values can enjoy the benefits of both sides

### 5.1. Experiment Setup

In order to comprehensively evaluate IMMAC, we consider different environments, various scenarios, diverse baselines, and ablations in this work.

#### 5.1.1. Environments

**Figure 2:**
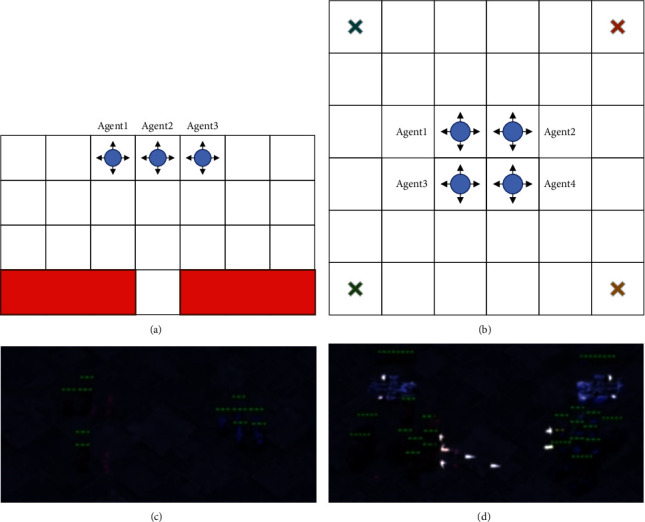
Examples of navigation tasks and SMAC scenarios. (a) Blocker. (b) Spread. (c) 5 m vs 6 m. (d) MMM2.

*(1) Cooperative Navigation*. As illustrated in Figures 2(a) and 2(b), Cooperative Navigation is a popular benchmark for multi-agent reinforcement learning. We adopt two variants used in [22] and refer them to Spread and Blocker, respectively. For both environments, it consists of a two-dimensional grid world and *n* independent agents. At each time step, agents can observe positions of themselves and need to decide to move toward one of the four directions. Agents would receive a team reward of −1 every moment before all of them arrive at the destinations. The global states including the positions of all agents are only available during training. In Spread environment, the shape of grid is set to 6 × 6. There are 4 landmarks located at four corners and each agent must navigate to a landmark different from others. In Blocker environment, the grid shape is set to 4 × 7. There are two blockers placed at bottom filed and three agents randomly initialized at the top row. The blockers that are responsible for blocking agents can move left or right in a deterministic rule. Agents must navigate to the bottom row while avoiding the blockers. In both environments, agent can observe its own position but know nothing about teammates, landmarks, and blockers, which makes the navigation tasks more difficult and requires a high level of communication.

*(2) StarCraft Multi-Agent Challenge (SMAC)*. Recently, SMAC [45] becomes a popular benchmark for evaluating the cooperative performance of RL agents. As illustrated in Figures 2(c) and 2(d), the environment consists a set of challenging scenarios based on StarCraft II, a well-known real-time strategy game. It provides interfaces to control a set of decentralized agents to fight against built-in AI. Concretely, each agent has a limited sight range and can only observe the information of adjacent units. The observation is typically represented as a feature vector consisting of relative position, distance, type, and health of observed units. The partial observability makes agents difficult to know whether the teammates and enemies out of sight range are still alive or not. The action space is discrete and different across scenarios. Typically, it consists of four types of available actions (move, attack, stop, and no − option). Moreover, the global states including coordinates and features of all agents are available only during training. The reward function is designed and shaped based on damaging, killing enemy units, or wining the combat. Overall, the SMAC environment is challenging due to the partial observability and complex state space. Furthermore, the scenarios are carefully designed and the fighting capacity of enemy is usually stronger. Consequently, it requires microtechniques and efficient cooperation to defeat the strong enemy. In this section, we select two hard and four super hard scenarios according to the taxonomy provided in [45]. The detailed classification and component of each selected scenario are shown in Table 1.

#### 5.1.2. Baselines and Ablations

- QMIX is a state-of-the-art centralized training and decentralized execution algorithm. It can achieve excellent performance in various multi-agent tasks, such as SMAC. We select it as the basic multi-agent reinforcement learning method and use it to represent a baseline without communication.
- QMIX with Tarmac is a variant to standard QMIX. The implementation considers Tarmac, a popular attentional communication protocol, where the attention weight is trained by downstream extrinsic rewards. We combine the attentional communication modules to QMIX framework and use it to represent a baseline with state-of-the-art extrinsic motivated communication.
- QMIX with IMMAC refers to the algorithm proposed in this paper.
- QMIX with Tarmac and IMMAC considers both extrinsic and intrinsic values to control communication. We aim to test whether the combination can enjoy the benefits of both worlds.

The detailed architectures and hyperparameters of baselines are similar to the one presented in [8, 19]. For fair comparison, the QMIX with Tarmac is implemented as 1-round communication. We adopt the straightforward way to combine Tarmac and IMMAC. Concretely, we summarize the attention weight of them and set *β*_*e*_=0.6 and *β*_*i*_=0.4. Moreover, the training paradigm is similar to [45]. We pause the training every 10^4^ time steps and run 32 test episodes for evaluation, and all results reported in following sections are averaged over 3 random seeds.

### 5.2. Intrinsic Value-Based Attention Mechanism

**Figure 3:**
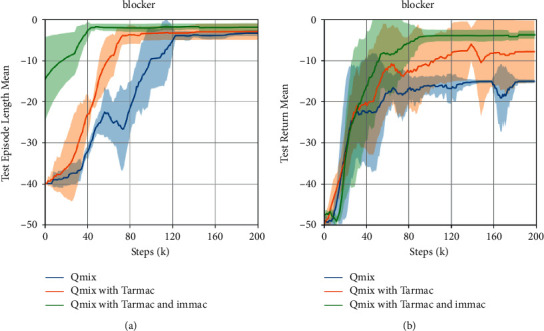
The learning curves of mean return in navigation tasks. The shaded area represents 95 % confidence intervals.

**Figure 4:**
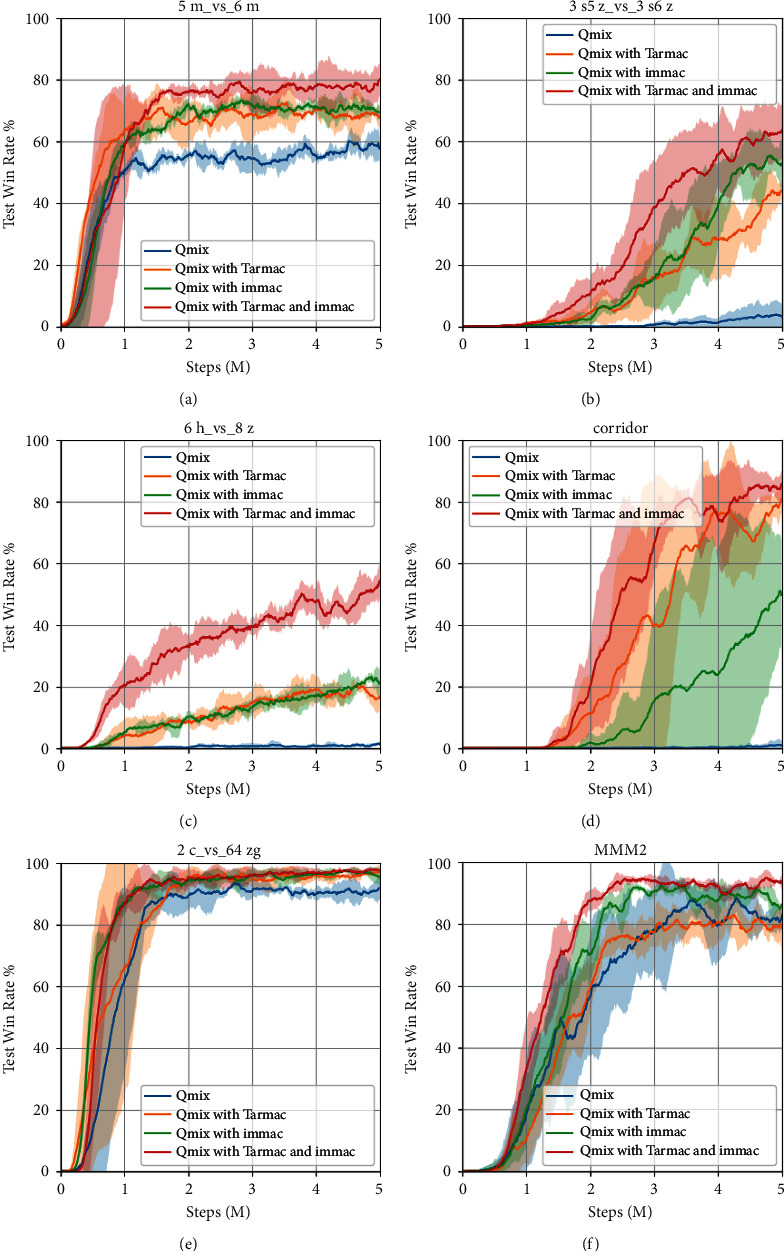
The learning curves of test win rates in SMAC scenarios. The shaded area represents 95 % confidence intervals.

In order to evaluate the performance of intrinsic attentional communication, we set *δ*=0 and consider full communication in this subsection. Then, we plot the test median return of navigation tasks in Figure 3. The results can preliminarily clarify our idea; that is, the communication can aid in cooperation, and then, combination of extrinsic and intrinsic motivation can further improve the communication efficiency. Furthermore, we present the median test win rate of the more challenging SMAC scenarios in Figure 4 and provide a detailed performance analysis.

At first, the performance of Tarmac and our IMMAC outperforms QMIX by a large margin in all environments. It shows that the efficient communication among agents can largely aid in cooperation in the partial observed scenarios. Concretely, the effective information sharing can help agent understand the global situation and make better decisions. Furthermore, we notice that our IMMAC can generate comparable performance with the state-of-the-art method Tarmac. It demonstrates our idea, in which the mechanism of intrinsic motivation can efficiently drive communication behaviors. Particularly, it shows that the intrinsic value can efficiently measure the importance of observed information then helps agent find helpful information from them without the help of extrinsic reward from environment so that our method enjoy better scalability. At last, we further notice that the combination of IMMAC and Tarmac can further improve the performance. It demonstrate that the proposed IMMAC is a good complement rather than a replace to the existing extrinsic motivated methods.

### 5.3. Intrinsic Value-Based Gating Mechanism

In practice, the attention model already equips agents with the ability to differentiate incoming messages at receiving end. It theoretically degrades the value of gating mechanism. However, the experiments in [9] indicate that the performance of Tarmac would significantly degrade when cutting off messages with smaller weights. Therefore, we would like to verify whether our algorithm is able to efficiently gate useless information while avoiding significant affect to performance. It is a key factor to apply IMMAC to real-world scenarios where communication resources are limited.

Concretely, we evaluate the performance of the following ablations:

- QMIX with IMMAC, which sets *δ*=0, it refers to a fully intrinsic motivated communication.
- QMIX with IMMAC, which sets *δ*=0.005.
- QMIX with IMMAC, which sets *δ*=0.008.
- QMIX with IMMAC, which sets *δ*=1, it degrades to QMIX without communication since the threshold is empirically larger than intrinsic values.

The communication rates and performance of gated IMMAC are presented in Tables 2 and 3, respectively. As shown in Table 2, we notice that the same threshold would generate different communication rates across scenarios, ranging from 34.1% to 58.8% and 27.1% to 45.1%. We find that the phenomenon is caused by the difference in observation-dependent intrinsic values. At first, the communication rate is jointly decided by intrinsic values and the threshold. Moreover, there is a subtle difference in observation spaces across scenarios and the observations collected by agents would also be different across episodes. Therefore, it is common that a difference of intrinsic values and communication rates exists across scenarios.

On the other hand, Table 3 indicates that the performance of our framework is not affected by the combination of gating mechanism. Although a part of messages is pruned, the performance of gated IMMAC is still obviously better than QMIX without communication. It even outperforms full communication by a small margin in 5m_*v*s_6m and 6*h*_*vs*_8*z* using only 30% to 50% communication resources. It demonstrates that full communication might not always aid in cooperation. There may be a part of useless messages, which cannot help or even degrade the decisions. The experiments in this subsection show that the intrinsic gating mechanism can effectively prune useless information sharing and once more demonstrate that the intrinsic values are efficient and promising to measure the importance of observed information.

Apart from evaluating the performance of gated IMMAC, we further provide an analysis why Tarmac does not work but IMMAC works well when introducing gating mechanism. Essentially, the Tarmac weights are trained through downstream loss and gradient of policy network. When combining with nondifferentiable gating modules, the attention weights (i.e., extrinsic values) would generate significant biases. Takes a scenario consisting of five agents as an example, assume that the output of Tarmac attention module is *α*_*t*_=(0.6, 0.1, 0.1, 0.1, 0.1). After gating, the weight becomes *α*_*t*_^*g*^=(0.6, 0,0,0,0). In practice, the gradient of policy network is computed and attention module is updated using *α*_*t*_^*g*^. However, the communication values of observations are evidently not zero and there is a bias of 0.1 for evaluating importance of observed information. On the other hand, IMMAC values are trained by an observation-dependent prediction problem and the gradient does not flow to gating module so that it would not be affected by the bias from gating mechanism. Furthermore, we examine the existing works related to extrinsic value-based control for multi-agent communication and find several works [5, 7, 44] model extrinsic values to gate messages, but whether the existing extrinsic values can scale to our framework with both attention and nondifferentiable gating models is still not clear to us. So, we only evaluate the performance of intrinsic value-based gating mechanism in this section and leave the combination of intrinsic and extrinsic value-based attention and gating in the future work.

## 6. Conclusions

We apply intrinsic motivation, a concept originated from psychology, to the literature of multi-agent communication. The objective of multi-agent communication is to improve the accuracy of decisions by information sharing. Hence, we conclude that how to evaluate the importance of observed information is the key to drive efficient communication behaviors. However, the existing works leverage extrinsic rewards but ignore the intrinsic values. We hold the view that intrinsic value can be a good complement to the existing works. Therefore, we propose a novel intrinsic motivated mechanism for multi-agent communication. Concretely, we adopt RND [11] to measure intrinsic novelty and uncertainty of observed information. Then, we apply an intrinsic value-based gating mechanism and an attention mechanism to the multi-agent communication framework. The gating mechanism can prune useless information and improve the efficiency of communication. The attention mechanism can help agent differentiate incoming messages and improve the accuracy of decisions. Finally, we extensively evaluate the performance of IMMAC. The results verify that intrinsic motivated communication is promising and it can generate better performance when combining with existing extrinsic motivated communication.

## Figures and Tables

**Table 1:** The detailed information of selected SMAC scenarios.

	*Allied units*	*Enemy units*
5m_vs_6m	5 marines	6 marines
2c_vs_64 zg	2 colossi	64 zerglings
3s5z_vs_3s6z	3 stalkers and 5 zealots	3 stalkers and 6 zealots
6h_vs_8z	6 hydralisks	8 zealots
Corridor	6 zealots	24 zerglings
MMM2	1 medivac, 2 marauders, and 7 marines	1 medivac, 3 marauders, and 8 marines

**Table 2:** Communication rate %.

	*δ*=0	*δ*=0.005	*δ*=0.008	*δ*=1
5*m*_*vs*_6*m*	100.0	56.5	44.8	0.0
3*s*5*z*_*vs*_3*s*6*z*	100.0	34.1	27.5	0.0
6*h*_*vs*_8*z*	100.0	58.8	45.1	0.0
Corridor	100.0	46.5	35.6	0.0
2*c*_*vs*_64*zg*	100.0	45.9	40.5	0.0
*MMM*2	100.0	35.9	27.1	0.0

**Table 3:** Test win rate of the last 25 × 10^4^ steps %.

	*δ*=0	*δ*=0.005	*δ*=0.008	*δ*=1
5*m*_*vs*_6*m*	70.7	76.5	77.8	58.2
3*s*5*z*_*vs*_3*s*6*z*	53.8	65.1	54.7	3.7
6*h*_*vs*_8*z*	21.7	29.4	27.2	1.2
Corridor	48.3	47.6	39.4	0.8
2*c*_*vs*_64*zg*	96.0	95.8	98.4	91.5
*MMM*2	86.9	84.8	92.4	82.2

## Data Availability

The data are trained based on an open MARL environment named SMAC and Cooperative Navigation, and the link of the environment is https://github.com/oxwhirl/pymarl.
